# Bmal1 suppresses cancer cell invasion by blocking the phosphoinositide 3-kinase-Akt-MMP-2 signaling pathway

**DOI:** 10.3892/or.2013.2381

**Published:** 2013-04-03

**Authors:** CHAN-HUN JUNG, EUN MI KIM, JONG KUK PARK, SANG-GU HWANG, SUNG-KWON MOON, WUN-JAE KIM, HONG-DUCK UM

**Affiliations:** 1 Division of Radiation Cancer Biology, Korea Institute of Radiological and Medical Sciences, Seoul 139-706, Republic of Korea; 2 Department of Biotechnology, Chungju National University, Chungju, Chungbuk, Republic of Korea; 3 Department of Urology, College of Medicine, Chungbuk National University, Cheongju, Chungbuk, Republic of Korea

**Keywords:** Bmal1, cancer invasion, tumor suppressor, circadian clocks, Bcl-w

## Abstract

Bmal1 is a core factor in the regulation of circadian rhythms. Previous studies have shown that Bmal1 suppresses tumor growth in cell culture and animal models and is down-regulated in certain types of cancer. The aim of the present study was to investigated whether Bmal1 influences the invasiveness of cancer cells. We demonstrated that knockdown of Bmal1 by RNA interference promoted cancer cell invasion, whereas its overexpression reduced cellular invasiveness. These effects were observed in lung cancer and glioma cells, and occurred regardless of p53 status. Therefore, it appears that Bmal1 suppresses the invasion of multiple cancer types in a p53-independent manner. Bmal1 knockdown-induced cancer cell invasion was accompanied by activation of the PI3K-Akt-MMP-2 pathway, and was prevented by inhibitors of PI3K, Akt or MMP-2. This suggests that Bmal1 suppresses cell invasion by blocking the PI3K-Akt-MMP-2 pathway. Since this invasion pathway is activated by the oncogene Bcl-w, we investigated whether Bmal1 affects the activity of Bcl-w. As expected, Bmal1 attenuated the ability of Bcl-w to promote MMP-2 accumulation and cell invasion, supporting the idea that Bmal1 antagonizes Bcl-w activity. Collectively, our data suggest that Bmal1 is a tumor suppressor, capable of suppressing cancer cell growth and invasiveness, and support the recent proposal that there is a tight molecular link between circadian rhythms and tumor formation/progression.

## Introduction

Metastasis of cancer cells is the most common reason for therapy failure. Although researchers have proposed a broad spectrum of mechanisms for cell migration and invasion, cancer therapeutics designed to block tumor progression by modulating these mechanisms have not yet proven effective in clinical trials. This may reflect the fact that cancer cells can operate different migration programs under different environmental conditions (1). Therefore, comprehensive understanding of the molecular and cellular underpinnings of cancer cell migration/invasion to better understand cancer metastasis and support the development of new treatment strategies is needed.

Circadian clocks, which are the body’s molecular time-keeping systems, form the basis for the daily rhythms of multiple biochemical, physiological and behavioral processes in most organisms (2,3). Importantly, substantial evidence suggests that dysfunctions of the circadian system are associated with pathological conditions, such as the formation and progression of cancer. For example, an increased risk of breast cancer was reportedly associated with female workers who were exposed to chronic disruptions of the sleep-wake cycle, such as flight attendants and rotating or permanent night-shift workers (4–6). Numerous other epidemiological studies have shown that perturbation of the normal circadian rhythm increases the risk of not only breast cancer, but also prostate, colorectal and endometrial cancers (7).

In mammals, the circadian system is regulated by a set of core clock factors, including Bmal1, Clock, casein kinase Iɛ, the cryptochromes (Cry1 and 2) and the periods (Per1-3), as well as supplementary regulators such as RORα and REV-ERBα (8–10). Per1 and Per2 are relatively well characterized in terms of their roles in cancer. They are reportedly downregulated in various types of human cancer (11–14), and Per2 gene-deficient mice exhibit an increased rate of lymphoma formation in response to ionizing radiation (15). At the molecular level, Per1 and Per2 are involved in the DNA damage response (16), and overexpression of either protein inhibits cancer cell growth and increases the apoptotic rate (16–18), supporting the notion that they participate in tumor suppression. Aside from these findings, however, there is little information regarding the molecular linkage between circadian rhythms and tumor formation/progression.

Bmal1 [brain and muscle aryl hydrocarbon receptor nuclear translocator (ARNT)-like] is a central clock factor that regulates the expression levels of the Cry and Per genes (19). Based on a recent report that downregulation of Bmal1 promotes tumor growth in cell culture and mice (20), we herein investigated whether Bmal1 also influences the invasiveness of cancer cells. The obtained data are presented in this study and the importance of our findings is discussed.

## Materials and methods

### Antibodies and inhibitors

Antibodies were purchased from the following institutions: anti-Bmal1 and anti-Akt from Santa Cruz Biotechnology (Santa Cruz, CA, USA); anti-phosphoinositide 3-kinase (PI3K) from Upstate Biotechnology (Lake Placid, NY, USA); anti-Bcl-w, anti-PTEN, and anti-phospho-Akt from Cell Signaling Technology (Danvers, MA, USA); anti-β-actin from Sigma-Aldrich (St. Louis, MO, USA); and anti-MMP-2 from Calbiochem (La Jolla, CA, USA). The synthetic inhibitors were obtained from Calbiochem.

### Cell culture, transfection and treatment

Human lung cancer cells (A549 and H1299) and glioma cells (U251) were cultured in RPMI-1640 and DMEM, respectively, supplemented with 10% heat-inactivated FBS. The Bmal1-expressing pCMV-SPORT6 vector (Thermo Fisher Scientific, Rockford, IL, USA), Bcl-w-expressing pcDNA3 vector (21), and siRNAs against Bmal1, Per3 and RORα (Ambion, Austin, TX, USA) were introduced into cells using Lipofectamine 2000 (Invitrogen, Carlsbad, CA, USA) according to the manufacturer’s protocol. All transfections were performed transiently, and transfectants were used for the indicated experiments following 40–48 h of the recovery.

### Western blot analysis

Cells were lysed on ice for 30 min in a buffer containing 20 mM Tris-HCl (pH 7.4), 100 mM NaCl, 0.5% NP-40, 0.1 mM Na_3_VO_4_, 50 mM NaF, 30 mM Na_4_O_7_P_2_ · 10 H_2_O and a protease inhibitor cocktail (GenDepot, Barker, TX, USA). To compare the levels of secreted MMP-2, cells were cultured for 24 h in serum-free medium and conditioned media were obtained. Proteins in the lysates or conditioned media were resolved by SDS-PAGE, and transferred to nitrocellulose filters (Millipore, Bedford, MA, USA) using an ECL Semi-Dry Transfer unit (Amersham Life Sciences, Uppsala, Sweden). The nitrocellulose filters were incubated with a blocking buffer (10% non-fat dry milk and 0.05% Tween in PBS) for 1 h and then incubated overnight with primary antibodies at 4°C. After incubation with secondary antibodies, peroxidase activity was assessed using a chemiluminescence-based detection system (Thermo Fisher Scientific).

### Invasion assay

The invasion assay was performed as previously described (21). Briefly, 0.2 ml of transfected cells (1–1.75×10^5^ cells/ml) in serum-free medium were seeded onto the upper surfaces of Matrigel-coated polycarbonate filters (BD Biosciences, Bedford, MA, USA) in a modified Boyden chamber (Corning Inc., Corning, NY, USA). The lower compartments of the chambers were filled with 0.6 ml of medium supplemented with 10% heat-inactivated FBS. After 24 h of incubation at 37°C and 5% CO_2_, the cells that had migrated to the lower surface of the filter were fixed, stained using a Diff-Quick kit (Fisher Scientific, Pittsburgh, PA, USA), and counted under a microscope.

### PI3K activity assay

The PI3K assay was conducted using a PI3K ELISA kit (Echelon Biosciences, Salt Lake City, UT, USA) according to the manufacturer’s protocol. Briefly, cells were lysed with ice-cold lysis buffer (20 mM Tris-HCl, pH 7.4, 137 mM NaCl, 1 mM CaCl_2_, 1 mM MgCl_2_, 1% NP-40, 1 mM PMSF and 0.1 mM sodium orthovanadate) for 20 min, and the cell lysates were subjected to immunoprecipitation with an anti-PI3K antibody. The immunoprecipitated PI3K was incubated with the PI(4,5)P2 substrate, and the generated PI(3,4,5)P3 was assayed by competitive ELISA.

### Statistical analysis

All experiments were carried out at least three times to obtain means and standard deviations as shown in the graphs in the figures. Results were analyzed for statistical significance using the Student’s t-test. Differences were considered significant at P<0.05.

## Results

### Bmal1 suppresses cancer cell invasion

**Figure 1:**
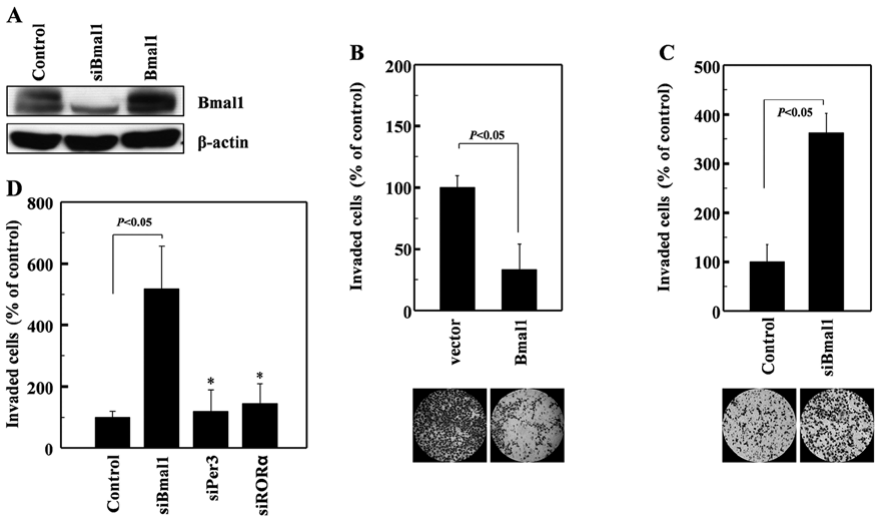
Bmal1 suppresses A549 lung cancer cell invasion. (A) Human A549 lung cancer cells were transfected with the empty (control) or Bmal1-expressing pCMV-SPORT6 vectors. Where indicated, the cells were alternatively treated with Bmal1-targeting siRNAs. After a 46-h incubation, the cellular levels of Bmal1 were analyzed by western blotting, using β-actin as a loading control. (B) The invasiveness of control and Bmal1-overexpressing cells was compared using Matrigel-coated membranes. (C) The invasiveness of cells transfected with either control or Bmal1-targeting siRNAs was compared. (D) A549 cells were treated with control siRNAs or those against Bmal1, Per3, or RORα, and cellular invasiveness was compared. The graphs show the means and SD from three independent experiments. ^*^P>0.05 vs. control siRNA.

To determine whether Bmal1 influences cellular invasiveness, we overexpressed Bmal1 in human A549 lung cancer cells (Fig. 1A). This resulted in a dramatic reduction in cellular invasiveness, as analyzed on Matrigel-coated polycarbonate filters (Fig. 1B). Conversely, the siRNA-mediated reduction in endogenous Bmal1 levels (Fig. 1A) promoted cell invasion (Fig. 1C). These findings suggest that Bmal1 suppresses cancer invasion. These effects were not mimicked by siRNAs against Per3 or RORα (Fig. 1D), suggesting that this invasion-suppressing activity is not common to all circadian factors.

**Figure 2:**
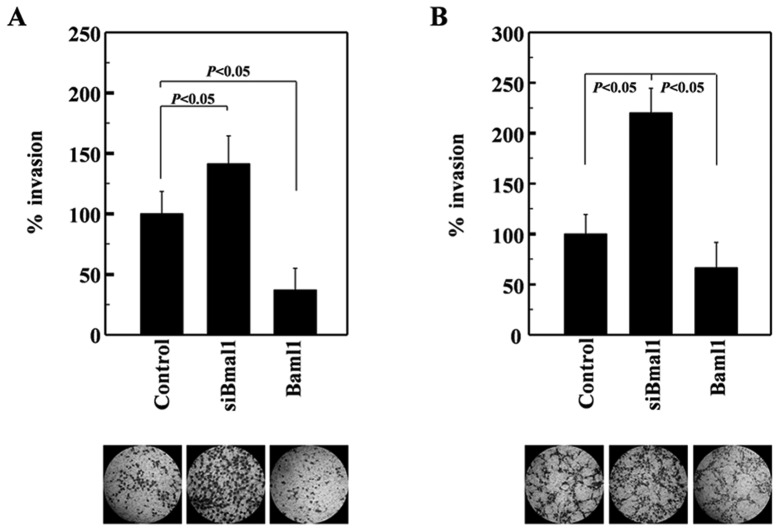
Bmal1 reduces the invasiveness of multiple types of cancer cells. (A) Human H1299 lung cancer and (B) U251 glioma cells were treated with Bmal1-targeting siRNAs or Bmal1-expressing vectors. After a 46-h incubation, cellular invasiveness was analyzed.

A549 cells express wild-type p53 (22). Given that this tumor suppressor is mutated in >50% of human tumors (23), we next investigated whether Baml1 suppresses the invasiveness of H1299 lung cancer cells, which were used as representative p53-null cells (24). The invasiveness of H1299 cells was also enhanced and suppressed by siRNA-mediated Baml1 knockdown and its overexpression, respectively (Fig. 2A). Therefore, Baml1 appears to suppress lung cancer cell invasion regardless of p53 expression. To determine whether Bmal1 exerts this function in cancer cells from other organs, we examined U251 glioma cells. The invasiveness of these cells was again increased and decreased by Bmal1 knockdown and overexpression, respectively (Fig. 2B), suggesting that Bmal1 suppresses the invasion of glioma cells. Together, these results suggest that Bmal1 reduces the invasiveness of multiple cancer types in a p53-independent manner.

### Bmal1 suppresses the PI3K-Akt-MMP-2 pathway

**Figure 3:**
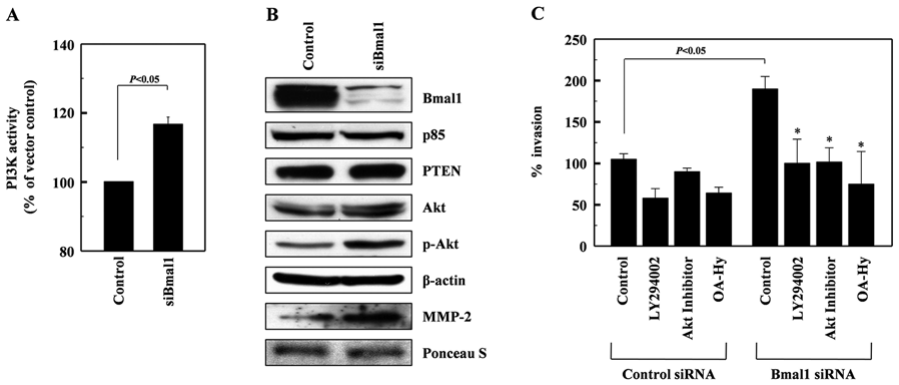
Bmal1 suppresses the PI3K-Akt-MMP-2 pathway. (A) Cell lysates from control or Bmal1-knockdown A549 cells were analyzed for PI3K activity by competitive ELISA. (B) The levels of Bmal1, the p85 subunits of PI3K, PTEN, Akt, phospho-Akt, and β-actin in the lysates were analyzed by western blotting. Alternatively, conditioned media were prepared using Bmal1-knockdown cells, and MMP-2 levels were analyzed by western blotting. Protein loading was verified by Ponceau S staining. (C) Control and Bmal1-knockdown cells were incubated in the presence or absence of LY294002 (5 μM), Akt inhibitor (5 μM), or OA-Hy (10 μM) for 24 h, and cellular invasiveness was compared. ^*^P<0.05 vs. Bmal1 siRNA control.

The PI3K-Akt-MMP-2 pathway is involved in promoting cell invasion under many experimental conditions (25–27). Here, we found that PI3K activity was elevated in Baml1-knockdown cells (Fig. 3A), while no change was evident in the levels of the p85 subunit of PI3K or PTEN, an endogenous inhibitor of PI3K (28) (Fig. 3B). This suggests that Baml1 knockdown induces PI3K activation. Bmal1 knockdown also elevated Akt phosphorylation and MMP-2 protein levels, suggesting that Baml1 may suppress the PI3K-Akt-MMP-2 invasion pathway. To confirm this, cells were treated with the Baml1-targeting siRNAs in the presence or absence of inhibitors for PI3K (LY294002), Akt (Akt inhibitor) and MMP-2 (OA-Hy). As expected, these inhibitors attenuated the Bmal1 siRNA-induced invasion of A549 cells (Fig. 3C), suggesting that Bmal1 reduces cellular invasiveness by suppressing the PI3K-Akt-MMP-2 pathway.

### Bmal1 antagonizes the invasion-promoting action of Bcl-w

**Figure 4:**
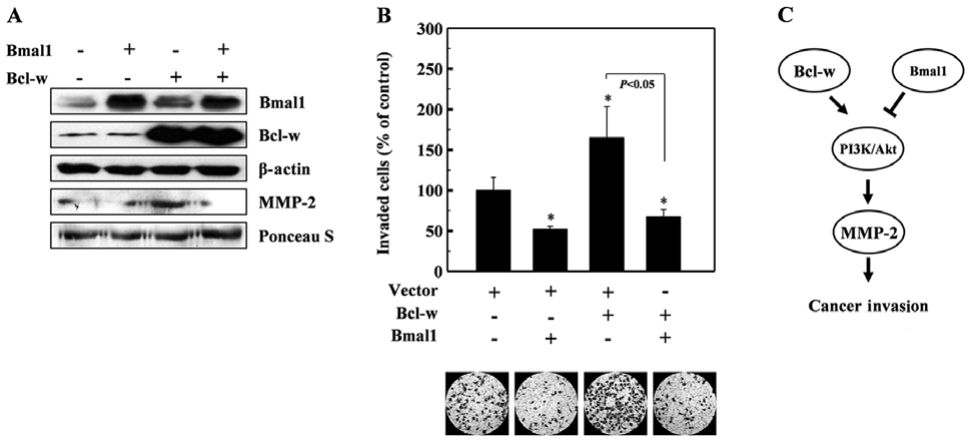
Bmal1 antagonizes the invasion-promoting action of Bcl-w. (A) H1299 cells were transfected with the indicated combinations of Bmal1- and Bcl-w-expressing vectors. After a 46-h incubation, cell lysates and conditioned media were prepared, and the levels of the indicated proteins were analyzed by western blotting. (B) The invasiveness of the transfectants was compared. ^*^P<0.05 vs. vector control. (C) Schematic model of the suppression of cellular invasiveness by Bmal1, via blockage of the PI3K-Akt-MMP-2 pathway. This contrasts with the ability of Bcl-w to promote cell invasion by activating the PI3K-Akt-MMP-2 pathway. Given that Bmal1 antagonizes Bcl-w-induced invasion, we speculate that Bmal1 and Bcl-w may compete to reciprocally regulate invasion.

Bcl-w is a pro-survival member of the Bcl-2 family of proteins (29), which are upregulated in various types of cancer cells (30,31). Bcl-w protects cells from apoptotic stimuli and also promotes the invasion of cancer cells by activating the PI3K-Akt-MMP-2 pathway (26,27). Therefore, we aimed to ascertain whether Bmal1 antagonizes the invasion-promoting action of Bcl-w. To accomplish this, we co-expressed Bmal1 and Bcl-w in lung cancer cells, and examined MMP-2 levels and cellular invasiveness. As reported previously (26,27), Bcl-w overexpression enhanced the levels of MMP-2 (Fig. 4A) and cellular invasiveness (Fig. 4B). However, both of these events were abolished by the co-expression of Bmal1 (Fig. 4A and B), suggesting that Bmal1 acts against Bcl-w to reduce cellular invasiveness.

## Discussion

In the present study, we demonstrated that Bmal1 suppresses cancer cell invasion. This was demonstrated by both Bmal1 overexpression and RNA interference experiments. This effect of Bmal1 was observed in lung cancer and glioma cells, indicating that Bmal1 reduces the invasiveness of multiple types of cancer. We believe that Bmal1 exerts this function by suppressing the invasion pathway that involves PI3K, Akt and MMP-2. This was initially suggested by the observation that Bmal1 knockdown increased the levels of PI3K activity, Akt phosphorylation, and MMP-2 protein, and was further confirmed by our observation that Bmal1 knockdown-induced cell invasion was blocked by inhibitors of PI3K, Akt, and MMP-2. Although certain circadian factors have been reported to regulate tumor growth and resistance (32), this is the first report that such a factor can also regulate cancer cell invasiveness.

Previous studies have shown that Bmal1 is downregulated in certain types of cancer (33) and suppresses tumor growth in cell culture and animal models (20). Together, these reports and our present results suggest that Bmal1 functions as a tumor suppressor. This view is further supported by our finding that Baml1 acts against the oncogene, Bcl-w, to prevent MMP-2 accumulation and cell invasion. As Bcl-w activates the PI3K-Akt-MMP-2 pathway, we propose that Bmal1 and Bcl-w may reciprocally regulate this invasion pathway (Fig. 4C).

The theory that Bmal1 acts as a tumor suppressor led us to examine the possible relationship between Bmal1 and p53, a well-characterized tumor suppressor that also suppresses cancer cell invasion (34). However, we found that knockdown of Bmal1 elevates the invasiveness of p53-expressing and p53-null lung cancer cells to almost equal extents. Therefore, Bmal1 appears to act as a tumor suppressor via a p53-independent mechanism, at least for the regulation of cellular invasiveness.

In conclusion, we showed that Bmal1 attenuates cancer cell invasion by suppressing the PI3K-Akt-MMP-2 pathway. This result supports the notion that there is a tight molecular link between circadian rhythms and tumor formation/progression.
